# Cannabinoid receptor 2 activation reduces intestinal leukocyte recruitment and systemic inflammatory mediator release in acute experimental sepsis

**DOI:** 10.1186/cc11248

**Published:** 2012-03-15

**Authors:** Christian Lehmann, Mandana Kianian, Juan Zhou, Inga Küster, Rieke Kuschnereit, Sara Whynot, Orlando Hung, Romesh Shukla, Brent Johnston, Vladimir Cerny, Dragan Pavlovic, Alexander Spassov, Melanie EM Kelly

**Affiliations:** 1 Department of Pharmacology, Dalhousie University, Halifax, Nova Scotia, Canada, B3H 2Y9; 2 Department of Anesthesia, Pain Management and Perioperative Medicine, Dalhousie University, Halifax, Nova Scotia, Canada, B3H 2Y9; 3 Department of Microbiology and Immunology, Dalhousie University, Halifax, Nova Scotia, Canada, B3H 2Y9; 4 Department of Anesthesia and Intensive Care, University Hospital Hradec Kralove, Faculty of Medicine Hradec Kralove, Charles University in Prague, Czech Republic; 5 Department of Anesthesia and Intensive Care, Ernst-Moritz-Arndt-University Greifswald, Germany; 6 Department of Orthodontics, Ernst-Moritz-Arndt-University, Greifswald, Germany

## Abstract

**Introduction:**

Cannabinoid receptor 2 (CB2R) expression is upregulated during sepsis. However, there are conflicting results regarding the effects of CB2R modulation in the hyperinflammatory phase of the disease. The aim of this study was therefore to investigate the effects of CB2R manipulation on leukocyte activation within the intestinal microcirculation in two acute experimental sepsis models.

**Methods:**

In the endotoxemia model we studied four groups of Lewis rats: controls, lipopolysaccharide (LPS), LPS + CB2R agonist HU308 (2.5 mg/kg), and LPS + CB2R antagonist AM630 (2.5 mg/kg). In the colon ascendens stent peritonitis (CASP)-induced sepsis model we also studied four groups: sham group, CASP and CASP + CB2R agonist (HU308, 2.5 or 10 mg/kg). Intravital microscopy was performed 2 hours following LPS/placebo administration or 16 hours following CASP/sham surgery to quantify intestinal leukocyte recruitment. Additionally, hemodynamic monitoring, histological examinations and measurements of inflammatory mediators were performed.

**Results:**

HU308 administration significantly reduced intestinal leukocyte adhesion in both acute sepsis models. The systemic levels of inflammatory mediators were significantly reduced by 10 mg/kg HU308 treatment in CASP animals.

**Conclusion:**

CB2R activation reduces leukocyte activation and systemic release of inflammatory mediators in acute experimental sepsis. Drugs targeting the CB2R pathway may have therapeutic potential in sepsis.

## Introduction

Cannabinoids from the plant *Cannabis sativa *have been widely used in medicine for over a millennium as anticonvulsant, analgesic, anti-emetic, anti-inflammatory and immunosuppressive drugs [1]. Cannabinoids, including both phytocannabinoids and synthetic cannabinoids, mediate their effect through binding to specific receptors, members of the G-protein-coupled receptor superfamily [2,3]. Cannabinoid-1 receptors are found throughout the central and peripheral nervous system, their major role being to modulate neurotransmitter release, whereas the cannabinoid-2 receptors (CB2Rs) are found mainly on immune cells and are known to play a role in immune responses and regulation of inflammatory processes [4,5]. The endogenous ligands for cannabinoid receptors are bioactive lipids called endocannabinoids. Collectively, endocannabinoids, their receptors and biosynthetic and degradative enzymes are referred to as the endocannabinoid system [1].

Sepsis and septic shock are the most frequent causes of death in surgical ICU patients [6]. The endocannabinoid system is upregulated during sepsis [7,8]. However, the role of the endocannabinoid system in the immune response is still not completely understood, particularly with specific regard to the complex disease progression of sepsis. To date, two studies have examined the impact of CB2R modulation on survival and other parameters in experimental sepsis induced by cecal ligation and puncture (CLP) using CB2R knockout mice. These studies reported opposing results. In one case, CB2R knockout mice following CLP-induced sepsis had a higher mortality and the administration of a selective CB2R agonist, GP1a, improved survival of wild-type mice [9]. In contrast, the second study demonstrated that CB2R knockout mice had a better survival from sepsis than did wild-type mice [10]. While these studies clearly demonstrate the importance of CB2R involvement in sepsis, they also underscore the requirement for further studies in order to clarify the mechanisms of CB2R-related modulation of inflammation in sepsis of varying severity and type.

The aim of our study was to investigate the effects of CB2R manipulation (activation or inhibition) in two different acute experimental sepsis models, given the conflicting studies of Tschöp and colleagues [9] and Csoka and colleagues [10]. We used 2 experimental sepsis models: endotoxemia and colon ascendens stent peritonitis (CASP)-induced sepsis. Endotoxemia was selected to study the effects of CB2R activation and inhibition in a standardized, acute model of systemic inflammation. CASP-induced sepsis was used to confirm the experimental results in a more clinically relevant model of sepsis.

Impairment of the intestinal microcirculation represents a key event in sepsis pathology [11,12]. The focus of our study was therefore the intestinal microcirculation. We evaluated leukocyte recruitment and functional capillary density in the intestinal microvasculature by intravital microscopy (IVM). Furthermore, we studied intestinal histology and plasma release of inflammatory mediators during experimental CB2R modulation.

## Materials and methods

### Animals

Male Lewis rats (250 ± 50 g) were purchased from Charles River (Wilmington, MA, USA), housed in chip-bedded cages and, prior to the experiments, acclimatized for 1 week in the air-filtered institutional animal care facility of the Faculty of Medicine at Dalhousie University, Halifax, Canada. Animals were kept on a 12-hour light/dark cycle, with the room temperature kept at 22°C and humidity at 55 to 60%. A standard diet of rodent chow and sterile drinking water were available *ad libitum*. The animal experiments were approved by the Dalhousie University Committee on Laboratory Animals (#11-072) and were performed in accordance with the standards and procedures set forth by the Canadian Council on Animal Care.

### Endotoxemia model

Animals were initially anesthetized with 54.6 mg/kg pentobarbital (Ceva Sante Animale, Montreal, QC, Canada) intraperitoneally and were supplemented with 20 mg/kg/hour pentobarbital intravenously during the experiment. Animals were then placed in a supine position on a heating pad maintaining a rectal body temperature of 37°C (98.6°F). Tracheostomy was performed to maintain airway patency, and animals breathed room air spontaneously. The left jugular vein and carotid artery were cannulated with polyethylene tubing (PE 50; Clay Adams, Sparks, MD, USA). Arterial blood pressure was recorded continuously (Monitor Model 66S; Hewlett Packard, Saronno, Italy).

Four groups (*n *= 9 mice per group) were examined. One group served as the control group. The other three groups received intravenously 20 mg/kg lipopolysaccharide (LPS) from *Escherichia coli*, serotype O26:B6 (Sigma-Aldrich, Oakville, ON, Canada): one group received LPS only; another group (LPS + HU308) received the CB2R agonist HU308 (2.5 mg/kg; Tocris Bioscience, Ellisville, MO, USA); and the final group (LPS + AM630) received the CB2R antagonist AM630 (2.5 mg/kg; Tocris Bioscience) intravenously after 15 minutes of endotoxemia (bolus injection). HU308 and AM630 were dissolved in dimethyl sulfoxide. Stock solutions were further diluted with saline (1:9). All control animals received dimethyl sulfoxide/saline as placebo. Laparotomy was performed at timepoint 1.5 hours with a stabilization period of 30 minutes before IVM at timepoint 2 hours.

### Colon ascendens stent peritonitis-induced sepsis model

The method has been described in detail previously [13]. Briefly, animals within the control group (*n *= 9) were laparotomized under general anesthesia (isoflurane) and the 16G stent was sutured outside of the intestine (sham operation). Three other groups of animals (*n *= 9 each) underwent the CASP procedure using the 16G stent inserted into the colon ascendens. One group remained untreated and the two other groups received HU308 (2.5 or 10 mg/kg intraperitoneally; Tocris Bioscience). Animals were recovered and IVM was performed 16 hours following CASP or sham procedure using the same protocol as for the endotoxemia experiments.

### Intravital microscopy

The abdomen was opened by a midline incision. A section of the distal small intestine from the ileocecal valve was placed carefully on a specially designed stage attached to the microscope [14]. During the entire *in vivo *microscopic procedure the intestine was superfused with thermostat-controlled (37°C/98.6°F) saline solution (0.9% sodium chloride; Hospira, Montreal, QC, Canada) to avoid drying and exposure to ambient air. The duration of each experiment, including induction of anesthesia, did not exceed 240 minutes. At the end of the experiments, the animals were euthanized by potassium chloride (EDM Chemicals, Gibbstown, NJ, USA).

IVM was performed using the following technical devices: an epifluorescent microscope (Leica DMLM, Wetzlar, Germany), a light source (LEG EBQ 100; Carl Zeiss, Jena, Germany), and a black and white monitor (Speco Technologies, Amityville, NY, USA). The images were transferred to the monitor by means of a CCD video camera (SIT 68; DAGE MTI, Michigan City, IN, USA) and were recorded using a videocassette recorder (DSR-25 DVCAM; Sony, Tokyo, Japan) for offline evaluation.

For investigation of leukocyte recruitment, leukocytes were stained *in vivo *by intravenous injection of 200 µl of 0.05% rhodamine 6G (1.5 ml/kg; Sigma-Aldrich), enabling visualization in the microvasculature. Six visual fields containing submucosal collecting venules (V1) over a length of at least 300 µm were observed and recorded for 30 seconds each.

Evaluation of all video sequences was carried out offline on a video monitor. We analyzed leukocyte adhesion (the number of leukocytes that stayed immobile for at least 30 seconds on an oblique, cylindrical endothelial surface; cells per square millimeter) in postcapillary venules (V3) and collecting venules (V1) of the intestinal submucosal layer.

### Inflammatory mediators

To quantify the plasma levels of TNFα, IL-1β, intercellular adhesion molecule, vascular cell adhesion molecule, RANTES and macrophage inflammatory protein-2, blood samples (1.0 ml) were drawn at the end of the CASP experiments and were immediately centrifuged at 5,000 rpm for 10 minutes to obtain plasma. The plasma was stored at -80°C until measurement. Cytokines and adhesion molecules were analyzed using a Procarta Multiplex Cytokine Assay kit from Affymetrix (Freemont, CA, USA) and a Bio-Plex instrument with Bio-Plex software (Bio-Rad, Mississauga, ON, Canada) according to the manufacturers' instructions.

### Histology

Tissue samples were collected from the intestine (LPS experiments only) and fixed in formalin (10%) for histological examination. The samples were stained with H & E and evaluated by light microscopy. The degree of histological injury in the small intestine was assessed using a grading scale: 0, normal histology; 1, slight disruption of the surface epithelium; 2, epithelial cell loss and injury at the villus tip; 3, mucosal vasocongestion, hemorrhage and focal necrosis with loss of less than one-half of villi; and 4, damage extending to more than one-half of villi [15].

### Statistical analysis

Results were analyzed using the software Prism 5 (GraphPad Software, La Jolla, CA, USA). All data are expressed as means ± standard deviation and were analyzed using one-way analysis of variance followed by the Bonferroni corrected Student's *t *test. Mean arterial pressure was analyzed by a two-way analysis of variance for repeated measures followed by Scheffé's test. Significance was considered *P *<0.05.

## Results

### Endotoxemia

**Figure 1:**
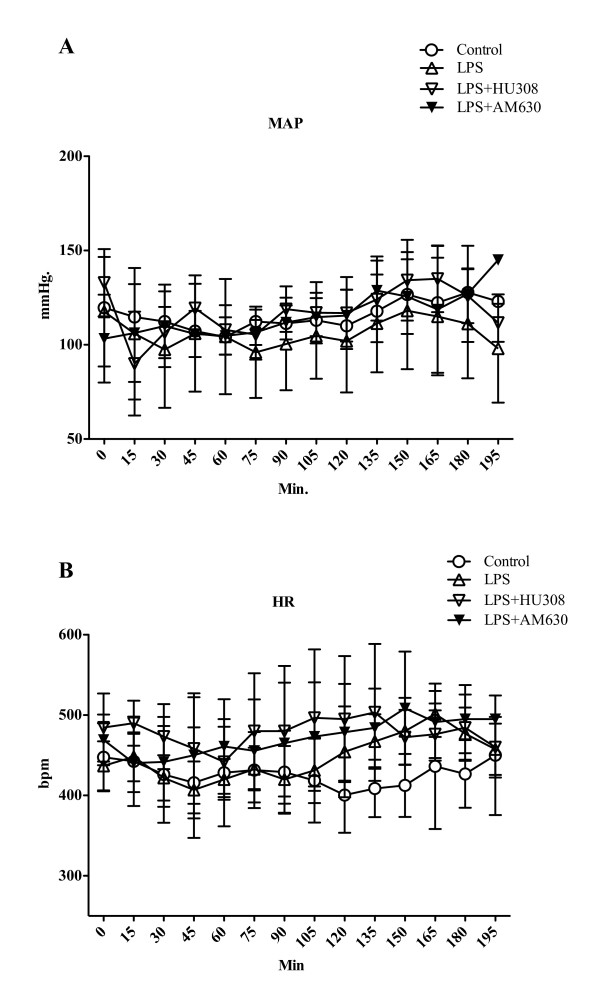
**Endotoxemia - mean arterial pressure and heart rate**. **(A) **Mean arterial pressure (MAP; mmHg) and **(B) **heart rate (HR; bpm): control group (*n *= 9); lipopolysaccharide (LPS), endotoxemia group (20 mg/kg LPS at time 0; *n *= 9); LPS + HU308, endotoxin plus cannabinoid receptor 2 (CB2R) agonist (*n *= 9); LPS + AM630, endotoxin plus CB2R antagonist (*n *= 9). Data presented as mean ± standard deviation.

The time course of mean arterial pressure (MAP) for all experimental groups is shown in Figure 1. For the endotoxin groups, MAP remained within the physiological range in the control, LPS and treatment groups during the observation period (Figure 1). Immediately after LPS administration, a slight drop in MAP was observed; however, at the time of IVM (120 minutes) all animals showed comparable MAP values.

**Figure 2:**
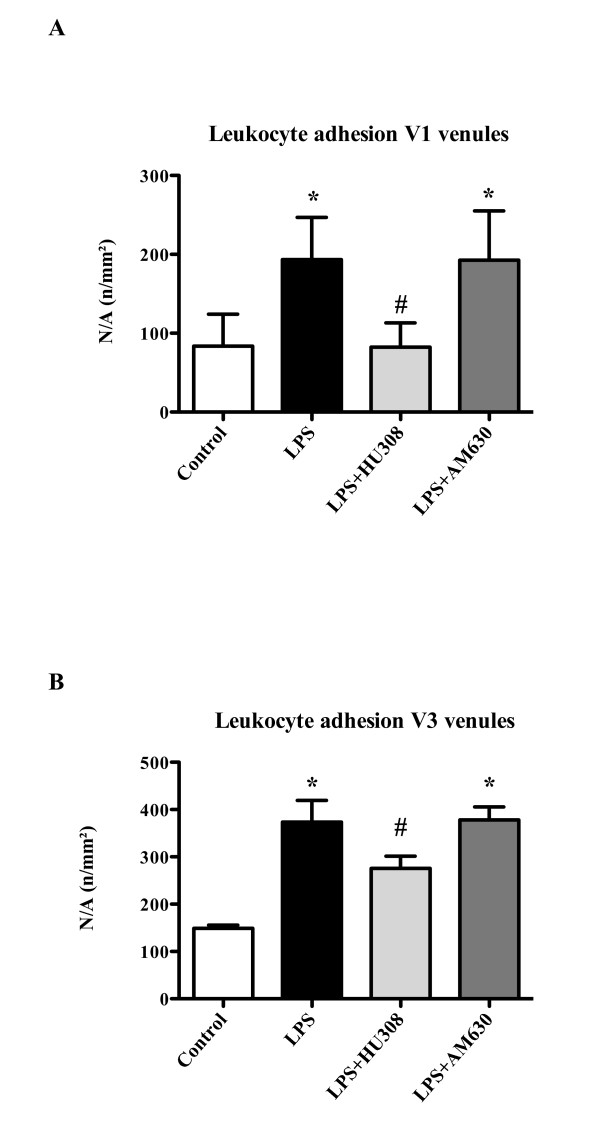
**Endotoxemia - leukocyte adhesion**. Leukocyte adhesion (n/mm^2^) in **(A) **collecting venules (V1) and **(B) **postcapillary venules (V3): control group (*n *= 9); lipopolysaccharide (LPS), endotoxemia group (20 mg/kg LPS at time 0; *n *= 9); LPS + HU308, endotoxin plus cannabinoid receptor 2 (CB2R) agonist (*n *= 9); LPS + AM630, endotoxin plus CB2R antagonist (*n *= 9). Data presented as mean ± standard deviation. N/A, numbers per area. *P <0.05 versus control. #*P *<0.05 versus LPS.

IVM measurements examining adherent leucocytes in intestinal submucosal venules are shown in Figure 2. Endotoxin challenge resulted in a 2.3-fold increase (*P *<0.0001) in the number of adherent leukocytes in V1 venules (Figure 2A). Treatment with the CB2R agonist HU308 decreased leukocyte adhesion by 57% (*P *<0.0001), while treatment with the CB2R antagonist AM630 showed no further increase of leukocyte recruitment.

Control animals showed a normal mucosa (Grade 0, according to Chiu and colleagues [15]). Short-time endotoxin challenge resulted in subepithelial space formation at villus tips and slight disruption of the surface epithelium. The histological score changed to Grade 1 to 2. The histological score for the CB2 agonist HU308-treated animals was Grade 0 (normal histology), and Grade 1 at a maximum for the tissue from the CB2 antagonist AM630-treated endotoxemic animals, showing a slight disruption of the surface epithelium (data not shown).

### Colon ascendens stent peritonitis-induced sepsis

**Figure 3:**
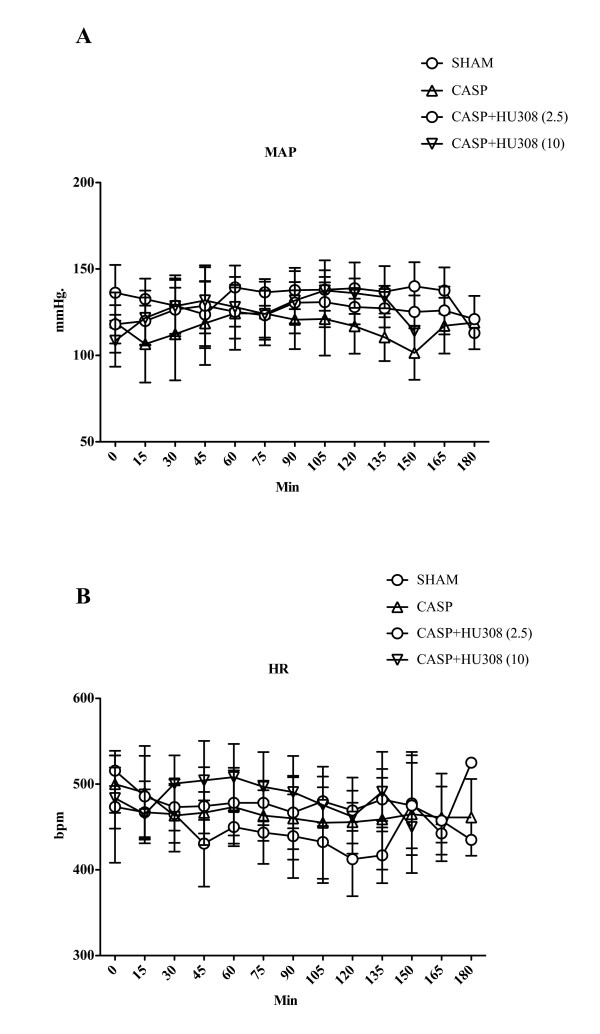
**Colon ascendens stent peritonitis-induced sepsis - mean arterial pressure and heart rate**. **(A) **Mean arterial pressure (MAP; mmHg) and **(B) **heart rate (HR; bpm): SHAM, control group (*n *= 9); colon ascendens stent peritonitis (CASP), sepsis group (*n *= 9); CASP + HU308 (2.5/10 mg/kg), sepsis plus cannabinoid receptor 2 agonist (2.5 or 10 mg/kg HU308; *n *= 9). Data presented as mean ± standard deviation.

As observed for the endotoxemia groups (see above), MAP for the CASP animals remained stable in the control (sham), CASP and treatment groups over the experimental observation period (Figure 3).

**Figure 4:**
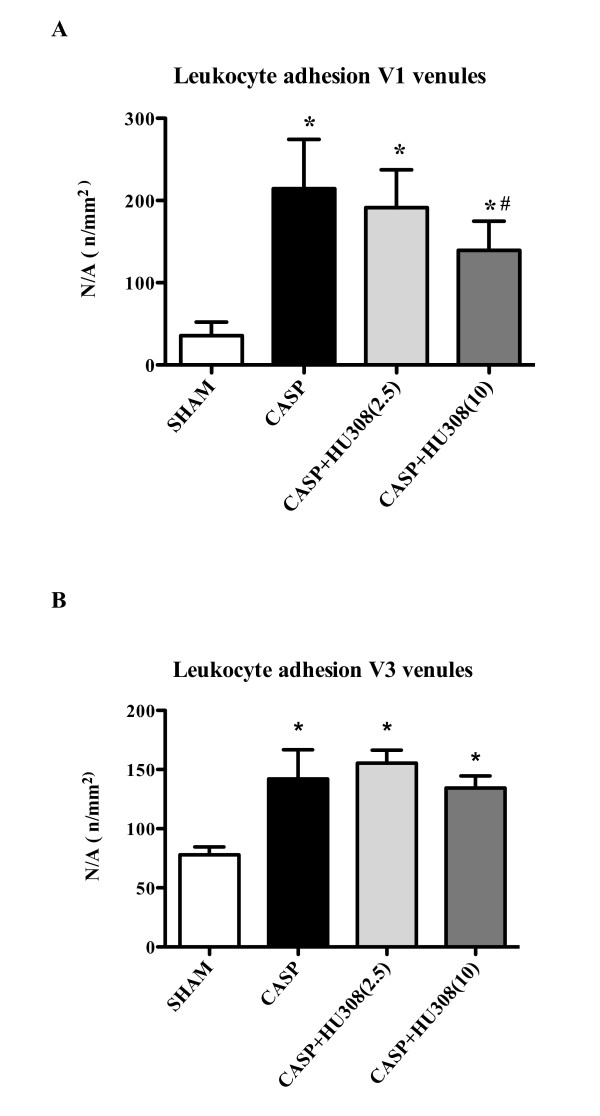
**Colon ascendens stent peritonitis-induced sepsis - leukocyte adhesion**. Leukocyte adhesion (n/mm^2^) in **(A) **collecting venules (V1) and **(B) **postcapillary venules (V3): SHAM, control group (*n *= 9); colon ascendens stent peritonitis (CASP), sepsis group (*n *= 9); CASP + HU308 (2.5/10 mg/kg), CASP plus cannabinoid receptor 2 agonist (2.5 or 10 mg/kg HU308; *n *= 9). Data presented as mean ± standard deviation. N/A, numbers per area. *P <0.05 versus SHAM. #*P *<0.05 versus CASP.

For CASP animals, a significant increase in the number of adherent leukocytes in V1 venules was detected after 16 hours of observation (Figure 4). Treatment with a low dosage of CB2R agonist HU308 (2.5 mg/kg) resulted in a slight (11%) but nonsignificant decrease of leukocyte adhesion. Administration of the higher dosage (10 mg/kg) HU308, however, resulted in a significant decrease of leukocyte recruitment compared with untreated endotoxin animals (35% decrease, *P *<0.001).

**Figure 5:**
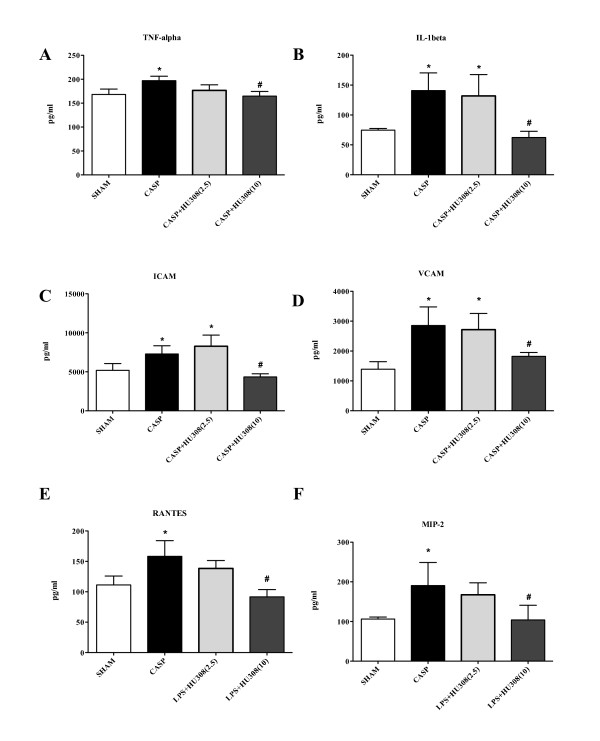
**Colon ascendens stent peritonitis-induced sepsis - inflammatory mediators (pg/ml)**. **(A) **TNFα, **(B) **IL-1β, **(C) **intercellular adhesion molecule (ICAM), **(D) **vascular cell adhesion molecule (VCAM), **(E) **RANTES, and **(F) **macrophage inflammatory protein-2 (MIP-2): SHAM, control group (*n *= 9); colon ascendens stent peritonitis (CASP), sepsis group (*n *= 9); CASP + HU308 (2.5/10 mg/kg), CASP plus cannabinoid receptor 2 agonist (2.5 or 10 mg/kg HU308; *n *= 9). Data presented as mean ± standard deviation. *P <0.05 versus SHAM. #*P *<0.05 versus CASP.

The CASP procedure resulted in significant elevated systemic levels of inflammatory mediators (Figure 5). Consistent with the observed reduction in adherent leucocytes (Figure 4), treatment with the high dosage (10 mg/kg) of HU308 significantly (*P *<0.05) reduced the levels of proinflammatory cytokines (TNFα, IL-1β), adhesion molecules (intercellular adhesion molecule, vascular cell adhesion molecule) and chemotactic factors (RANTES, macrophage inflammatory protein-2).

## Discussion

The main finding of our study was that CB2R activation reduced leukocyte recruitment within the intestinal microvasculature in two distinct acute experimental sepsis models.

In the first model (endotoxemia) we observed a significant increase in the number of adhering leukocytes in the intestinal microvasculature caused by LPS challenge. We found that the CB2R agonist HU308, given at a dosage of 2.5 mg/kg intravenously, significantly reduced the number of adherent leukocytes in submucosal V1 and V3 venules. The fluorescence dye Rhodamine 6G used for intravital imaging stains all leukocytes, so the specific cell types could not be further determined [16]. However, because of the high percentage of neutrophils in the bloodstream, this subpopulation is expected to be the most important cell fraction in this regard. In our LPS experiments, we observed no significant effects of the CB2R antagonist AM630 on LPS-induced leukocyte adhesion. LPS-induced leukocyte adhesion was probably already at its maximum and therefore could not be further exacerbated by CB2R block.

As CB2R inhibition by AM630 showed no impact on leukocyte recruitment in our (endotoxemia) model, we used only the agonist HU308 in the CASP-induced sepsis experiments. The CASP acute sepsis model showed stable macrohemodynamics (no septic shock). However, the agonist dosage used in the LPS endotoxemia experiments was not sufficient to produce significant effects on leukocyte adhesion in the CASP model; an increase in the dosage of HU308 to 10 mg/kg was required to obtain the same extent of reduction of intestinal leukocyte adhesion. In support of the results from our CASP model, Ni and colleagues also showed in a more clinical-related experimental inflammation model (autoimmune encephalomyelitis) that the cannabinoid 1/cannabinoid 2 receptor agonist WIN 55212-2 was only able to attenuate leukocyte-endothelial interactions in the cerebral microcirculation if given at a higher dosage (10 mg/kg) [17].

Taken together, these findings indicate that the effect of CB2R activation on inflammation is dose dependent and varies according to the experimental conditions. The conflicting survival results of the experimental sepsis studies reported in CB2R knockout mice [9,10] may thus reflect differences in the severity of the CLP-induced sepsis model used in these studies. For example, CB2R activation was only beneficial in more severe sepsis, comparable with endotoxemia used in our study. However, in less severe CLP models, inefficient clearance of bacterial pathogens could lead to prolongation of the infectious process and resultant death. Attenuation of inflammation would thus prove more beneficial for survival in a severe model of CLP, but could lead to increased mortality in a moderate model of CLP [18].

In support of this hypothesis, our inflammatory mediator data provided evidence that the CASP-induced sepsis model generated an acute proinflammatory cytokine and adhesion molecule profile. Administration of (the higher dose of) the CB2R agonist HU308 decreased the levels of proinflammatory, soluble adhesion molecules and chemotactic factors, consistent with an anti-inflammatory effect of CB2R activation in this sepsis model.

TNFα is a key proinflammatory cytokine involved in mediating endotoxemia and sepsis [19]. Inhibition of TNFα production therefore has a high therapeutic potential in managing sepsis. Several studies have now reported that endocannabinoids can modulate the release of proinflammatory mediators via CB2R-related pathways. For example, the endocannabinoids anandamide and 2-arachidonylglycerol were observed *in vitro *to inhibit the production of TNFα released from LPS-treated rat microglial cells [20]. Another study showed that 2-arachidonylglycerol inhibits TNFα release from LPS-treated murine macrophages both *in vitro *and *in vivo *[21]. The involvement of CB2R in regulating the release of TNFα was reinforced in several studies using β-caryophyllene, a selective CB2R agonist. β-Caryophyllene significantly inhibited LPS-stimulated TNFα expression in human peripheral blood [22], and a recent *in vitro *study by Bento and colleagues showed that, in addition to TNFα, treatment with β-caryophyllene also reduced release of IL-1β and IFNγ by LPS-stimulated macrophages [23]. In keeping with these results, our study also found a significant reduction of the systemic IL-1β release in animals with CASP-induced sepsis treated with the CB2R agonist HU308. Furthermore, we also observed a reduction in the levels of soluble adhesion molecules by CB2R activation in CASP-induced sepsis. Several groups have shown that the levels of soluble adhesion molecules are correlating with sepsis severity and outcome [24]. The observed decrease in the levels of (soluble) adhesion molecules is in line with our IVM findings of attenuated leukocyte recruitment within the intestinal microvasculature by CB2R activation. Studies using other CB2R agonists (for example, JWH133 and HU309) during hepatic ischemia/reperfusion injury demonstrated that CB2R activation also resulted in a reduction of TNFα and macrophage inflammatory protein-2 levels, and tissue expression of adhesion molecule intercellular adhesion molecule-1 [25,26]. These beneficial effects of CB2R agonists could be attenuated by CB2R genetic deletion or inhibition.

The expression of inflammatory mediators is regulated by various signaling pathways, including NF-κB activation, which are affected by CB2R modulation [27]. For example, Rajesh and colleagues demonstrated that CBR activation attenuates TNFα-triggered NF-κB and RhoA activation [28]. Furthermore, noladin ether, a stable analog of the endocannabinoid 2-arachidonylglycerol, inhibited the nuclear translocation of NF-κB that leads to the arrest of the cell cycle and inhibition of growth of prostate carcinoma cells [29]. Taken together, these studies suggest that endocannabinoid signaling, and specifically CB2R modulation, is able to alter signaling pathways that regulate production of inflammatory mediators.

## Conclusion

The data reported in this study support the involvement of CB2R signaling in leukocyte activation during sepsis. We have shown that CB2R activation has anti-inflammatory actions in two acute experimental models of severe sepsis in rats. Drugs targeting the CB2R may have therapeutic potential in the treatment of inflammatory diseases, such as severe sepsis.

## Key messages

• Administration of the CB2R antagonist HU308 significantly reduced intestinal leukocyte recruitment in two acute sepsis models.

• The systemic levels of cytokines and adhesion molecules were significantly reduced by 10 mg/kg HU308 treatment in peritonitis-induced sepsis.

• The data reported in this study support the involvement of CB2R signaling in leukocyte activation during systemic inflammation/sepsis.

• Drugs targeting the CB2R pathway may have therapeutic potential in sepsis.

## Abbreviations

CASP: colon ascendens stent peritonitis; CB2R: cannabinoid receptor 2; CLP: cecal ligation and puncture; H & E: hematoxylin and eosin; IFN: interferon; IL: interleukin; IVM: intravital microscopy; LPS: lipopolysaccharide; MAP: mean arterial pressure; RANTES: Regulated upon Activation: Normal T-cell Expressed: and Secreted; TNF: tumor necrosis factor; V1: collecting venule; V3: postcapillary venule.

## Competing interests

The authors declare that they have no competing interests.

## Authors' contributions

CL, MK and MEMK conceived of the study, analyzed data, and drafted the manuscript. MK, IK and RK carried out intravital microscopy. MK carried out cytokine and adhesion molecule measurements. JZ and BJ supervised the experimental procedures. VC, DP and AS made substantial contributions to the conception and design of the study. SW, OH and RS were involved in revising the manuscript critically for important intellectual content. All authors read and approved the final manuscript.
